# Economic Recession and the Risk of Cancer: A Cohort Study From Eastern Finland

**DOI:** 10.2188/jea.JE20200595

**Published:** 2022-08-05

**Authors:** Rand Jarroch, Behnam Tajik, Tomi-Pekka Tuomainen, Jussi Kauhanen

**Affiliations:** University of Eastern Finland, Institute of Public Health and Clinical Nutrition, Kuopio, Finland

**Keywords:** economic recession, socioeconomic position, cancer, population-based, cohort study

## Abstract

**Background:**

Little is known about the role of economic recessions in the risk of cancer. Therefore, we evaluated the impact of the severe economic recession in Finland from 1991–1994 on the incidence of all cancers and cancer subtypes among a middle-age and older population.

**Methods:**

From the Kuopio Ischemic Heart Disease Risk Factor Study (KIHD), a population-based sample of 1,620 women and men aged 53–73 years were examined from 1998–2001. The cancer-free participants completed a questionnaire on the possible impact of the 1990s recession in Finland on their lives. Incident cases of cancer were obtained through record linkage with the Finnish Cancer Registry. Cox proportional hazards regression was used to estimate hazard ratios (HR) of incident cancer events after adjusting for possible confounders.

**Results:**

A total of 1,096 cancer-free participants had experienced socioeconomic hardships due to the recession at the baseline. During 20 years of follow-up, 473 participants developed cancer. After adjustment for age, baseline socioeconomic position, and lifestyle factors, the risk of all cancers was 32% higher among men who experienced socioeconomic hardships compared to those who did not (HR 1.32; 95% confidence interval [CI], 1.00–1.74, *P* = 0.05). Prostate-genital cancer was 71% higher among men with hardships (*n* = 103, HR 1.71; 95% CI, 1.06–2.74, *P* = 0.02). No association was observed between socioeconomic hardships and subsequent risk of total or any subtype of cancer among women.

**Conclusion:**

The 1990s economic recession was associated with increased risk of all cancers, especially prostate-genital cancer among Finnish middle-age and older men, but no association with cancer was observed in women.

## INTRODUCTION

Cancer is the second leading cause of death worldwide.^1^ Several biological and environmental risk factors of cancer have been identified. Socioeconomic position (SEP) and the change of SEP across the life-course are likely to influence cancer risk factors, thus associating with cancer incidence and mortality.^2^ Economic recessions often change the SEP in part of the population,^3^ causing various social and financial disadvantages, which are termed as socioeconomic hardships.^4^

Most of the previous studies on recessions and health have investigated the physical and mental health only among those who have become unemployed during recessions.^5^^,^^6^ The global financial crisis in 2008, particularly, awakened the scientific community to the possible effects that recessions may have on cancer. Research findings, however, are still inconsistent.^5^^,^^7^ Some studies have found an increase in cancer mortality among men and women since the onset of recession.^8^^–^^13^ Some, in contrast, have reported a decline in cancer mortality^14^ and lower cancer incidence during recession.^15^^,^^16^ To our best knowledge, there are no prior studies examining possible longer-term impacts of recessions on subsequent cancer incidence.

Finland experienced a sudden, rapid, and exceptionally severe collapse of its economy in the early 1990s following many years of strong economic growth during the 1980s.^3^ This economic downturn affected the country for many years after and caused unemployment rates to peak at 19.8% in 1996, while it had been 5.2% prior to the recession in 1989.^17^ Although the dramatic changes in the Finnish economy likely affected the population health in many ways, few studies have investigated the details, and most studies have focused only on all-cause mortality during the recession period.^17^^,^^18^ Therefore, we wanted to investigate whether the socioeconomic hardships that resulted from the 1990s severe recession would suggest longer-term impacts on the Finnish population health. Specifically, the study aimed to examine the post-recession incidence of cancer in a population-based sample of middle-age and older women and men in Eastern Finland by comparing those who had and those who had not been exposed to socioeconomic hardships during the recession.

## METHODS

### Study population

We performed a prospective analysis among the participants from the Kuopio Ischemic Heart Disease (KIHD) Risk Factor Study.^19^ KIHD is an ongoing prospective population-based study, which initially started in 1984 to investigate the different risk factors of cardiovascular disease (CVD), atherosclerosis, and related outcomes in middle aged men in Eastern Finland. Later, it extended to study other non-communicable diseases. The first cohort consisted of 1,166 men who were 54 years old, enrolled in 1984–1986. To extend the study to cover more age groups, additional sampling and baseline examination was performed in 1986–1989, and it included groups of 42-, 48-, and 60-year old men, in addition to original 54-year olds (*N* = 1,516). A total of 920 women aged 53–73 years participated in KIHD for the first time from 1998–2001.^20^

**Figure 1.**
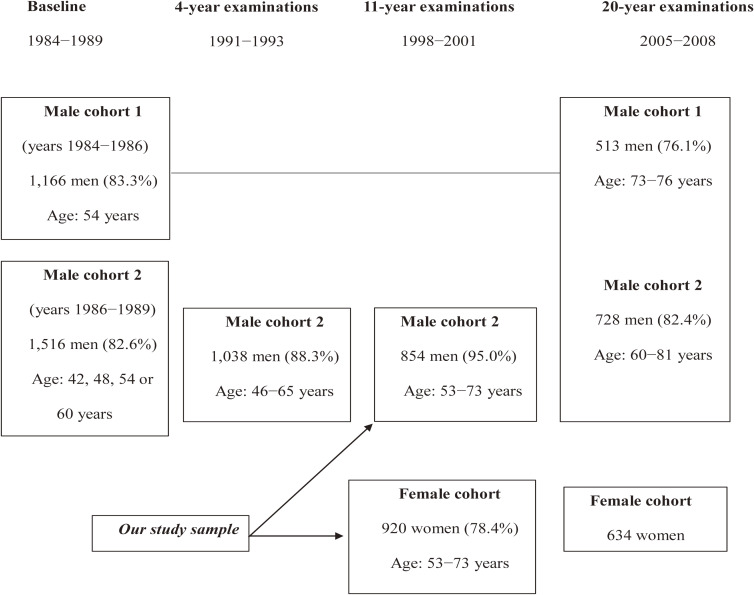
Timeline of the Kuopio Ischemic Heart Disease Risk Factor Study. The percentages in parentheses indicate the proportion of eligible participants that participated in the study visits.

Our study is based on 1,774 middle-age and older women and men who were examined from 1998–2001. The women cohort comprised of 920 women (78.4% of the 1,173 eligible) aged 53–73 years. The men cohort comprised 854 men aged 53–73 (85.6% of those who participated in KIHD from 1986–1989) (Figure 1).

The KIHD protocol was approved by the Research Ethics Committee of the University of Kuopio and complies with Declaration of Helsinki. All the subjects signed a written informed consent.

**Figure 2.**
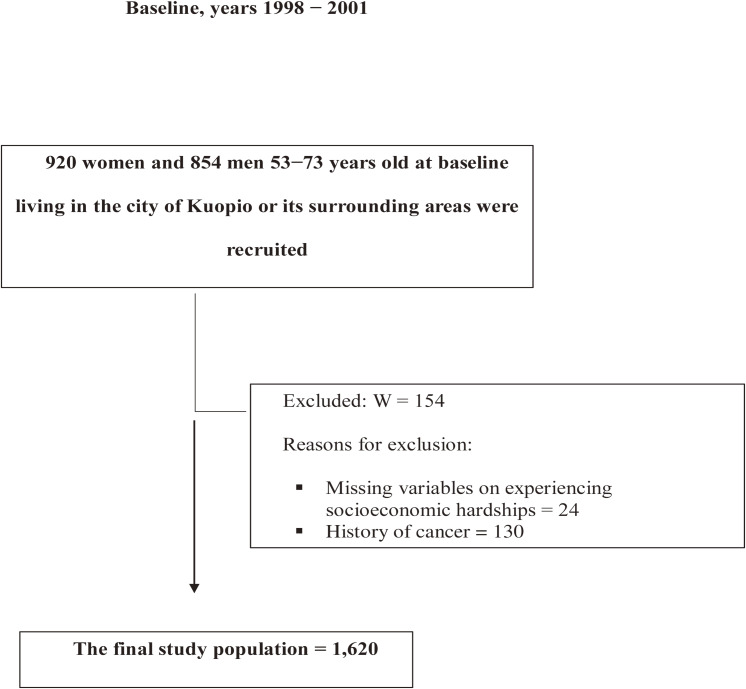
Study population.

From the analyses, we excluded participants with missing data on experiencing socioeconomic hardships (*n* = 24) as well as participants with a prior history of cancer (*n* = 130). After the exclusions, 1,620 women and men who were free of cancer were included in the study sample (Figure 2). The baseline examinations took place from 1998–2001, and the possible exposure to the recession had happened years before, in the time period of 1991 through 1994.

### Measurements

#### Baseline socioeconomic position (SEP)

Participants completed questionnaires on their socioeconomic background. As education and marital status were two SEP components that were not affected by the recession, we used them to adjust for baseline SEP. The other frequently used SEP variables were already implemented in the inquiry of participants’ experiences during the recession. Education was measured in number of years. Marital status was categorized into four groups: married or living with a partner, not married, separated or divorced, and widowed.

#### Defining socioeconomic hardships

A new and comprehensive measure was introduced to estimate the overall hardships caused by the recession. Participants were asked whether Finland’s economic recession from 1991–1994, which peaked 4–9 years before the baseline examination, had influenced their personal or family economic and psychological situation. The detailed questionnaire included questions on income reduction, unemployment, bankruptcy and loss of property. Original responses were grouped into two categories: participants who did and participants who did not experience personal or immediate family-related socioeconomic hardships because of the recession. Any hardship counted as an exposure, whether one or more of them were listed by the participant.

#### Other risk factors

A questionnaire was used to check smoking status.^20^ Alcohol consumption was assessed using the Nordic Alcohol Consumption Inventory for drinking behavior over the previous 12 months with a structured quantity-frequency method.^21^ A trained nurse checked and completed the questionnaires during interviews. Physical activity was assessed using the 12-Month Physical Activity questionnaire to record the frequency, average duration, and intensity of the most common physical activities of Finnish middle-aged people.^22^ Fruits, vegetables, and berries consumption was calculated based on 4-day food records at the time of blood sampling. Body mass index (BMI) was calculated by dividing weight in kilograms by the square of height in meters.^23^

#### Ascertainment of cancer follow-up events

Incident cancer cases were derived from the Finnish Cancer Registry (FCR), which is a national population-based digital registry covering all of Finland with no lost cancer cases since 1953. The cancer diagnoses for the registry are determined and reported by the secondary or tertiary health care units (hospitals, pathological and hematological laboratories, physicians, and dentists).^24^ Our study cohort was linked with the FCR data using the 11-digit personal identity code mandatory to every resident of Finland. Outcome was assessed annually through re-linkage with the registry using these personal identity codes. All cancer events that occurred between the baseline examination and the end of 2017 were included.

### Statistical analysis

The univariate associations between experiencing socioeconomic hardships and baseline socioeconomic, lifestyle, and clinical characteristics were assessed using means and linear regression for continuous variables and Chi^2^ independency test for categorical variables to explore bivariate relationships.

No wash-out period for incident cancer cases was necessary, since the economic recession had already occurred in Finland in the early 1990s; in other words, more than 5 years before the study subjects participated in the study and the cancer follow-up started.

Hazards ratios (HRs) for the risk of cancer according to socioeconomic hardships exposure binaries were estimated using Cox regression models. The analysis was performed on three different levels: first, by investigating the HRs for the risk of all cancer events among all participants, then by stratifying according to gender, and finally, by investigating the HRs for the risk of each cancer subtype among each gender. The category that did not experience any hardships was considered as the reference. The criteria for selecting confounders were based on established risk factors for cancer or on associations with exposures or outcomes in the present analysis.

Two models were used to adjust for potential confounders in the prospective analyses. Model 1 adjusted for age (years). Model 2 additionally adjusted for sociodemographic variables of education (years) and marital status (married or living as a couple, not married, separated or divorced, widowed), smoking status (yes/no), alcohol intake (g/week), physical activity (hour/year), mean of fruits, vegetables and berries consumption (g/4 days) and BMI (kg/m^2^).

Missing values within each of the covariates (<0.5%) were replaced by the cohort mean. All *P*-values were two-sided (α = 0.05). All analyses were conducted with the SPSS statistical software (version 27, SPSS Inc., Chicago, IL, USA).

## RESULTS

### Baseline characteristics

A total of 1,096 (68%) women and men reported experiencing socioeconomic hardships during the 1990s recession. Baseline characteristics of the participants are presented in Table 1 according to the two exposure categories. Participants who experienced hardships due to the recession were more likely to be younger, having been unemployed at some time in earlier years before the recession, and more likely to smoke as compared to participants who did not experience any hardships. The exposed group also had on average lower income and higher BMI (Table 1).

**Table 1.** Baseline characteristics according to the level of socioeconomic hardships

**Level of the socioeconomic hardships due to the economic recession**			
**Variables**	Did not experience socioeconomic hardships (*n* = 524) [294 women and 230 men]	Experienced socioeconomic hardships (*n* = 1,096) [536 women and 560 men]	*P*-value
**Age, years**	64.1 (6.3)	61.9 (6.4)	≤0.001
Women	64.7 (6.2)	62.0 (6.4)	≤0.001
Men	63.4 (6.4)	61.8 (6.4)	0.002
**Education, years**	9.8 (3.7)	9.5 (3.3)	0.07
Women	9.7 (3.5)	9.7 (3.2)	0.76
Men	9.9 (4)	9.3 (3.4)	0.03
**Income, €/year**	17,451 (12,155)	15,864 (11,101)	0.01
Women	14,341 (7,864)	13,854 (8,046)	0.4
Men	21,387 (15,180)	17,764 (13,112)	0.01
**Marital status**			0.75
Married/Living as a couple	75%	74.5%	
Not married	6.9%	6.6%	
Divorced/Separated	6.7%	10.3%	
Widowed	11.5%	8.6%	
Women			0.57
Married/Living as a couple	66.7%	63.8%	
Not married	8.8%	8%	
Divorced/Separated	8.2%	13.6%	
Widowed	16.3%	14.6%	
Men			0.79
Married/Living as a couple	85.7%	84.8%	
Not married	4.3%	5.2%	
Divorced/Separated	4.8%	7.1%	
Widowed	5.2%	2.9%	
**Unemployment year**	1989 (7.7)	1987 (15)	0.03
Women	1989 (8.9)	1987 (15.8)	0.10
Men	1989 (5.9)	1987 (14.2)	0.15
**Current smoker, %**	10.5%	15%	0.01
Women	5.4%	10.3%	0.02
Men	17%	19.5%	0.41
**Alcohol intake, g/w**	47.6 (85.8)	50.3 (109.9)	0.63
Women	20.1 (39.5)	18.4 (37.1)	0.53
Men	82.8 (112.2)	80.7 (143)	0.84
**BMI,^b^ kg/m^2^**	27.6 (4.2)	28 (4.6)	0.05
Women	27.7 (4.9)	28.6 (5.2)	0.01
Men	27.3 (3.1)	27.4 (3.8)	0.82
**Physical activity, hours/year**	536.6 (439.7)	540.8 (471.6)	0.86
Women	590.4 (467.3)	630.1 (541.1)	0.29
Men	467.8 (392)	455.4 (374.1)	0.68
**CRP, mg/L**	2.9 (4.4)	3.0 (5.3)	0.84
Women	3.1 (4.7)	3.1 (5.2)	0.85
Men	2.7 (4)	2.9 (5.3)	0.52

BMI, body mass index; CRP, C-reactive protein.

Results being presented are mean (SD) for continuous variables and *n* (%) for categorical data.

The mean age for participants with socioeconomic hardships was 61.9 (standard deviation [SD], 6.4) years compared to 64.1 (SD, 6.3) years of those who had no hardships (*P* ≤ 0.001). Men who experienced hardships had less education (9.3; SD, 3.4 years) compared to men who did not (9.9; SD, 4 years) (*P* = 0.03). In women, there were no significant differences in the educational level between exposed and non-exposed.

### Association of the socioeconomic hardships and the incidence of cancer

During mean follow-up of 20 years, the total number of new cancer cases observed in the cohort was 473, of which 216 were among women and 257 among men. After adjustment for age (model 1), the risk of any cancer was 20% higher among participants (men and women combined), who experienced socioeconomic hardships compared to those who did not (HR 1.20; 95% confidence interval [CI], 1.00–1.50, *P* = 0.07). Further adjustments for baseline SEP and lifestyle variables slightly strengthened the association (multivariate-adjusted HR 1.22; 95% CI, 1.00–1.50, *P* = 0.05). When stratified by gender, the increased risk of any type of cancer was observed only among men who had experienced socioeconomic hardships (HR 1.28; 95% CI, 1.00–1.69, *P* = 0.08 for model 1 and HR 1.32; 95% CI, 1.00–1.74, *P* = 0.05 for model 2). However, the *P*-value for gender interaction was 0.45, actually not suggesting clear gender-based interaction.

In further investigation of cancer subtypes, we found a 71% higher risk of prostate-genital cancer among men who had experienced socioeconomic hardships as compared with those who did not (*n* = 103, HR 1.56; 95% CI, 1.00–2.49, *P* = 0.05 for model 1 and HR 1.71; 95% CI, 1.06–2.74, *P* = 0.02 for model 2) (Table 2). Again, no associations were seen between being hit by socioeconomic hardships and the later incidence of cancer subtypes in women (data not shown).

**Table 2.** Hazard ratios for cancer events according to the level of socioeconomic hardships

**Level of socioeconomic hardships due to the economic recession binaries**			
**Variables**	Did not experience socioeconomic hardships (reference group) (*n* = 524)	Experienced socioeconomic hardships (*n* = 1,096)	*P*-value
***N of cases of cancer, %***	142 (27.1)	331 (30.2)	
*Women*	74 (25.2%)	142 (26.5%)	
*Men*	68 (29.6%)	189 (33.8%)	
HR model 1^*^			
All Participants		1.20 (1.00–1.50)	0.07
Women		1.06 (0.79–1.41)	0.69
Men		1.28 (1.00–1.69)	0.08
Prostate-Genital Cancer in Men		1.56 (1.00–2.49)	0.05
HR model 2^*^			
All Participants		1.22 (1.00–1.50)	0.05
Women		1.06 (0.79–1.41)	0.71
Men		1.32 (1.00–1.74)	0.05
Prostate-Genital Cancer in Men		1.71 (1.06–2.74)	0.02

Values are hazards ratios (95% confidence interval).

Model 1^*^: adjusted for age.

Model 2^*^: adjusted for model 1 plus education, marital status, smoking status, alcohol intake, physical activity, consumption of fruit, vegetables and berries, and body mass index.

## DISCUSSION

In our population-based follow-up study of 1,620 middle age and older people from Eastern Finland, the risk of all cancers combined, and especially prostate-genital cancer, was increased among men who had gone through socioeconomic hardships during the economic recession, which had occurred around 5 years before our study baseline and the start of cancer follow-up. No increased risk of gender-specific or any other cancer was observed in those women who also had experienced socioeconomic hardships.

The impact of macroeconomic crises on population health in general, and on cancer in particular, is still unclear and controversial.^7^ Some studies have assessed the association of economic recessions and the incidence of all cancer events^15^^,^^16^ and all cancer mortality^11^ in women and men. Other studies focused on certain cancer subtypes mortality,^8^ including gender-specific cancers.^9^ Short-term cancer mortality during recessions and unemployment have been the most frequently used measures in studies on recessions and cancer.^6^

In our study on the 1990’s recession in Finland, women and men who experienced hardships due to recession were more likely having been unemployed already at a younger age, before the recession happened. They had on average a lower income at baseline, compared to those who did not experience any hardships in recession. Individuals without stable income or other financial security, and those who already had met with frequent unemployment spells, may have been further affected by the economic slump, and not only those salary earners, who lost their jobs during the recession.

In some cases, socioeconomic hardships felt by our study participants did not directly result from themselves losing their jobs. As they were asked to report also the unemployment of other family members, the whole family situation may have comprised the hardship, at least psychologically.

Psychological stress, on molecular and cellular level, can be one possible mediating process between hardships and cancer risk. Stress induces hypothalamic–pituitary–adrenal axis and sympathetic nerve system dysfunction, as well as cytokines imbalance, thus contributing to the development of cancer.^25^ Following this line of thought, our findings might suggest women being more resilient than men in coping with the economically induced psychological stress. This, in turn, would partly explain why recession-related hardships did not show increased cancer risk among women who were exposed to them. Some previous research supported this hypothesis. A study on the 1990s recession effect on Finnish population found an increase in mortality, but only among highly educated male workers, and one explanation offered was the higher psychological stress in this group.^17^ On the other hand, in our study the men who showed higher incidence of cancer in the follow-up were on average less educated and had lower income. This denotes complexity as to what role psychological stress actually could play as a possible mediating mechanism. More research is definitely needed to unravel the etiologic details.

In general, it is well-established that men have higher cancer mortality rates than women^26^ and are more likely to develop, for example, colorectal cancer.^27^ In addition to the possible biological differences between men and women regarding cancer, the attitudes and behaviors towards cancer screening programs seem to differ. The decision to attend cancer screening is difficult for many men, with a frustrating chance of leading to further screenings.^26^

During recessions, austerity might affect screening programs in some countries, and as a consequence more cancer deaths and severe cases are expected to follow later.^11^ However, this was not the case during the 1990s recession in Finland, where screening programs continued with high participation rates and austerity measures on healthcare were not widely applied.^5^ Although Avendano et al^17^ argued that Finns were more resilient to recessions because of generous social security benefits and unemployment insurance, this may not apply to health care needs. Keskimaki^28^ found a more than 10% increase in acute hospitalization rates among Finns aged 25–74 during the 1990s recession.

Harmful health effects of recessions might take several years to become evident. Therefore, longer-term follow-ups are needed in epidemiological studies.^29^ Most research on recession and health outcomes, especially regarding mortality, have used only short follow-ups during the actual recession period. In fact, findings are often explained as a result to cut-offs in health expenditure and decrease in access to healthcare.^10^^,^^30^ Generally, earlier studies do not consider the possibility of more direct mechanisms in disease development during economic crises. As the current SARS-CoV-2 (Covid-19) pandemic will trigger numerous studies on the economic crisis and its impact on population health,^31^ new viewpoints are hopefully opened. To put it shortly, more research is still needed to better understand the role of macroeconomics on health and on disease etiology. It can be already agreed that health policies should give more attention to preparatory investments and proactive measures in public health programs, education, counselling and other human resource-based health activities.

### Strengths of the study

Our epidemiological study is based on a regionally and ethnically representative population-based sample. The follow-up time of almost 20 years can be considered sufficiently long in these age groups. The comprehensive and reliable nationwide system of digital registers that was utilized in our study, covers all data on hospital discharge diagnoses, causes of death, and incident cases of cancer in Finland. Therefore, the outcome measure in our follow-up study can be considered reliable.

The majority of previous studies on recessions and cancer have used unemployment as the only measure of socioeconomic hardships. Instead, we used a detailed questionnaire to draw a broader estimate on how the participants were overall affected by the recession, including, but not limited to, unemployment. Finally, the large dataset of the KIHD study allowed for a broad range of well-validated measures to adjust for in our models predicting incident cancer. Most previous studies have focused on cancer mortality only, with much more limited set of covariates to control for.

### Limitations of the study

Since our study was based on an ethnically homogenic population of middle-aged and older Finnish women and men, we cannot necessarily generalize the results to other ethnic groups and countries. On the other hand, at least the other Nordic countries share fairly similar demographic characteristics and same type of social and welfare system. Still, the results may not be generalized even in Finland to women and men other age groups than to those we studied.

To avoid over-adjustment, we included only two covariates describing socioeconomic position (SEP); namely, education and marital status. This was justified simply by the fact that the other widely used SEP components, income and occupation, were already included in the participants’ responses on whether they were affected by the recession or not. The size of our cohort was large enough to allow only analysis of the most common cancers, therefore some potentially important associations may have been missed. Finally, while our study showed an association between socioeconomic hardships and subsequent cancer risk, any causal inferences regarding cancer pathophysiology should be treated with extreme caution.

### Conclusion

Our study suggests that economic recessions may pose gender-specific cancer risks to middle-age and older Finnish men, but not necessarily to women. The 1990s severe economic recession in Finland and the subsequent socioeconomic hardships it imposed on men were associated with an increased risk of developing especially prostate-genital cancer during the 20 years of follow-up.
